# Supplementary figures and images for: Loss of tumor suppressor TMEM127 drives RET-mediated transformation through disrupted membrane dynamics
Source: eLife. 2024 Apr 30;12:RP89100. doi: 10.7554/eLife.89100 (PMC11060712; doi:10.7554/eLife.89100)

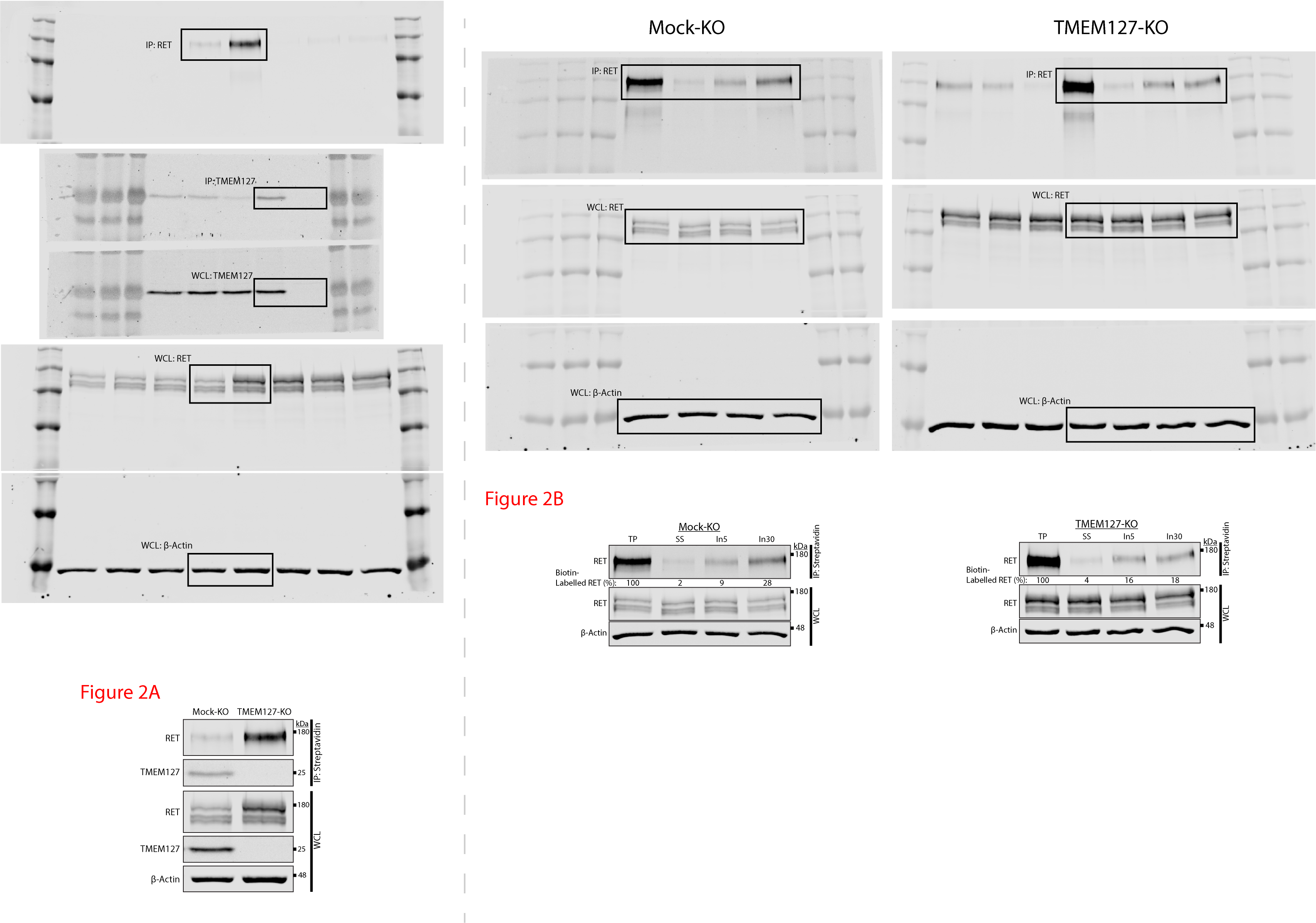

Supplement: Figure 2—source data 3. [file elife-89100-fig2-data3.zip › Figure 2 - Source Blots.tif]

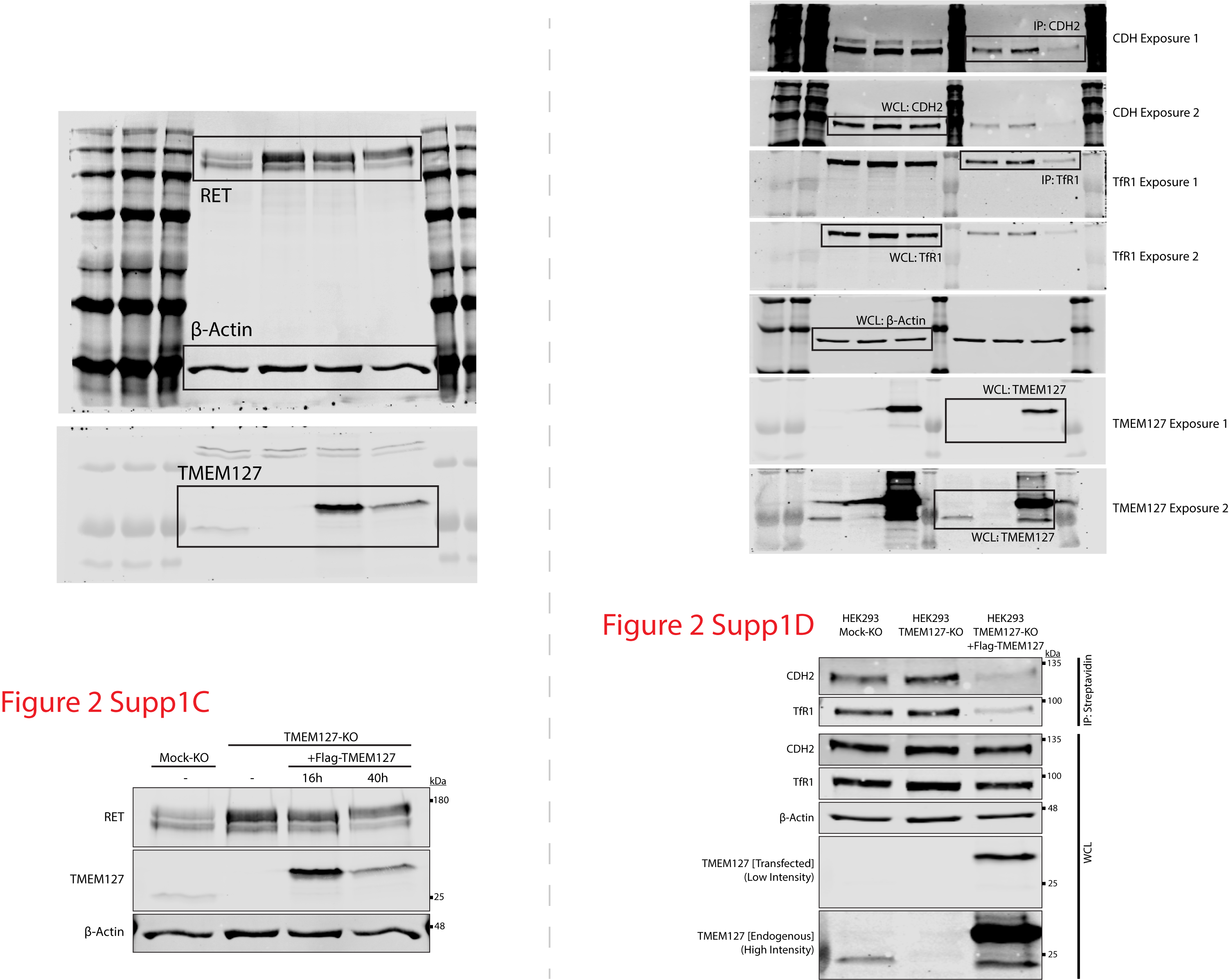

Supplement: Figure 2—figure supplement 1—source data 3. [file elife-89100-fig2-figsupp1-data3.zip › Figure 2 Supplement 1 - Source Blots.tif]

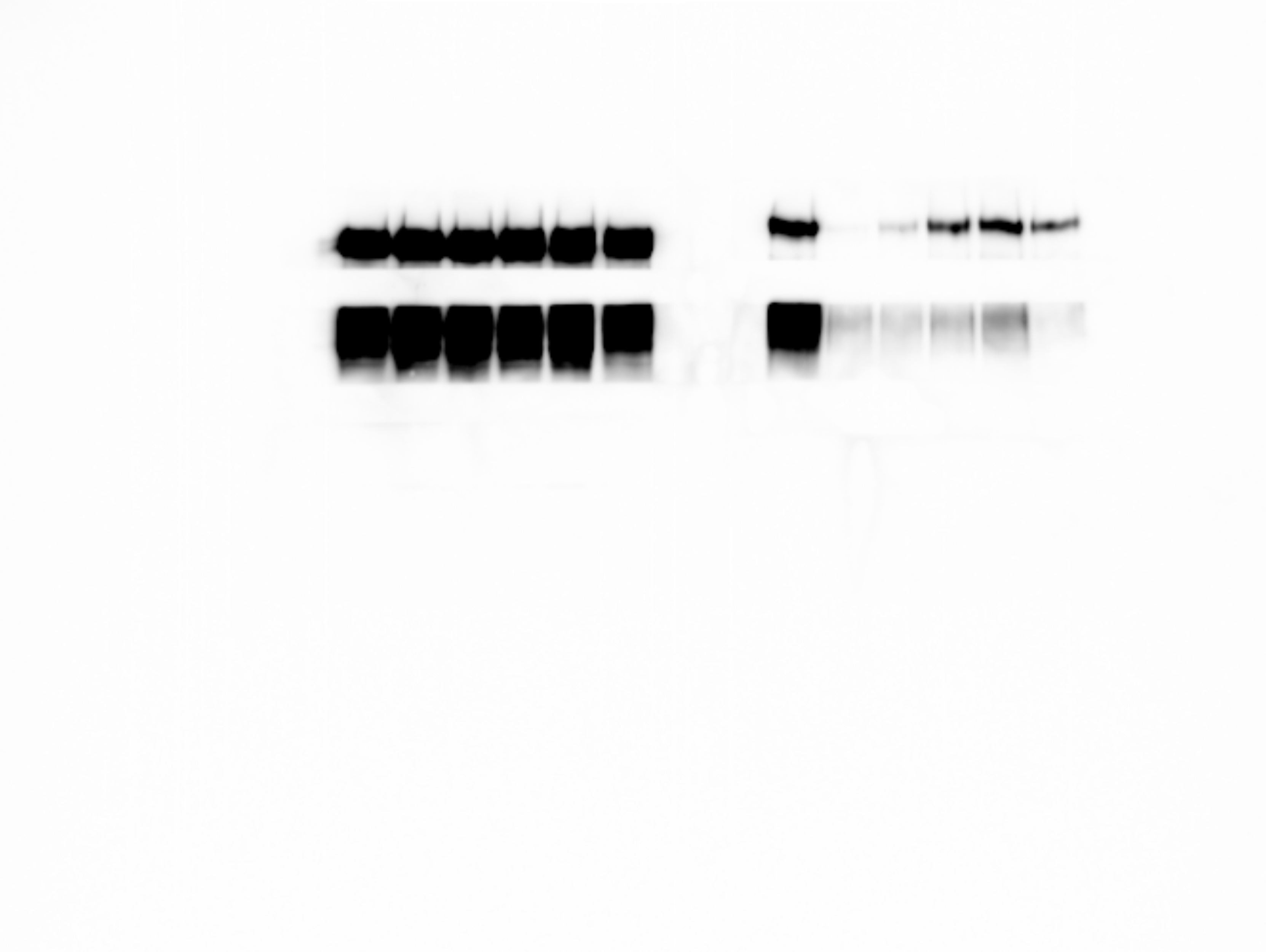

Supplement: Figure 2—figure supplement 2—source data 2. — Image Studio Lite files contain a histogram of signal and individual image brightness was adjusted for each protein of interest and shown separately. [file elife-89100-fig2-figsupp2-data2.zip › Figure 2 Supp2 Mock-KO IP_RET.tif]

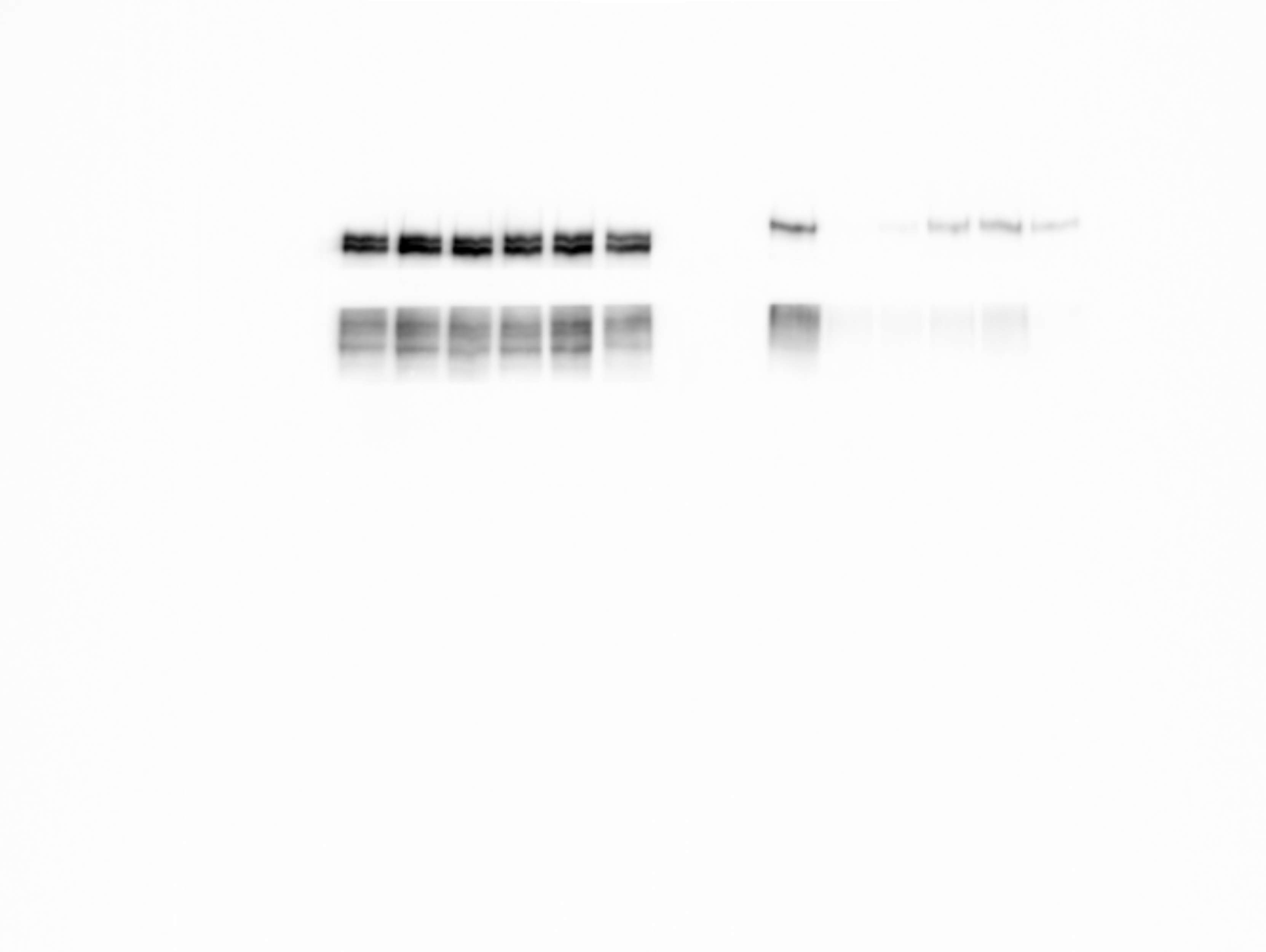

Supplement: Figure 2—figure supplement 2—source data 2. — Image Studio Lite files contain a histogram of signal and individual image brightness was adjusted for each protein of interest and shown separately. [file elife-89100-fig2-figsupp2-data2.zip › Figure 2 Supp2 Mock-KO WCL_RET.tif]

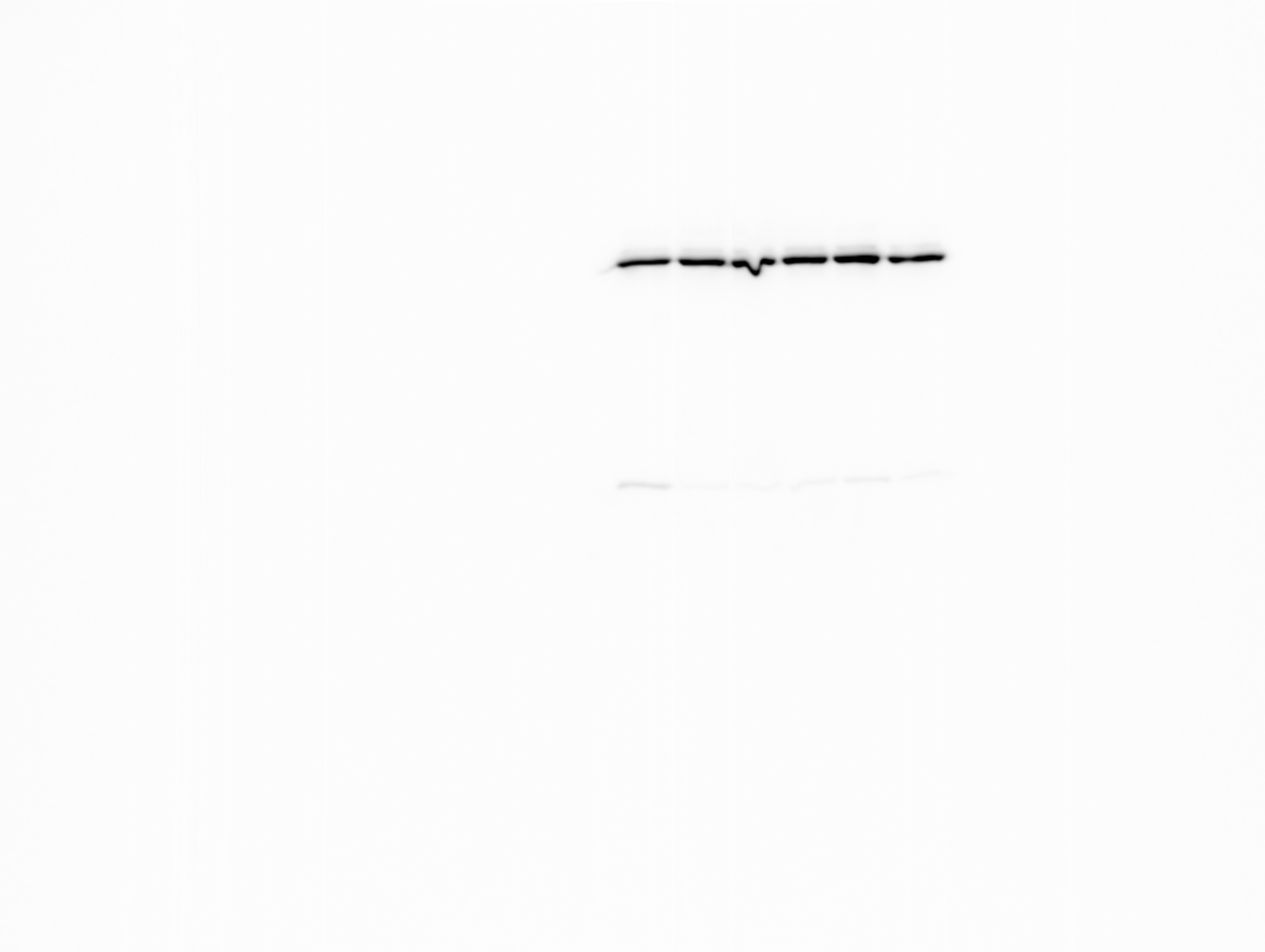

Supplement: Figure 2—figure supplement 2—source data 2. — Image Studio Lite files contain a histogram of signal and individual image brightness was adjusted for each protein of interest and shown separately. [file elife-89100-fig2-figsupp2-data2.zip › Figure 2 Supp2 Mock-KO WCL_Tubulin.tif]

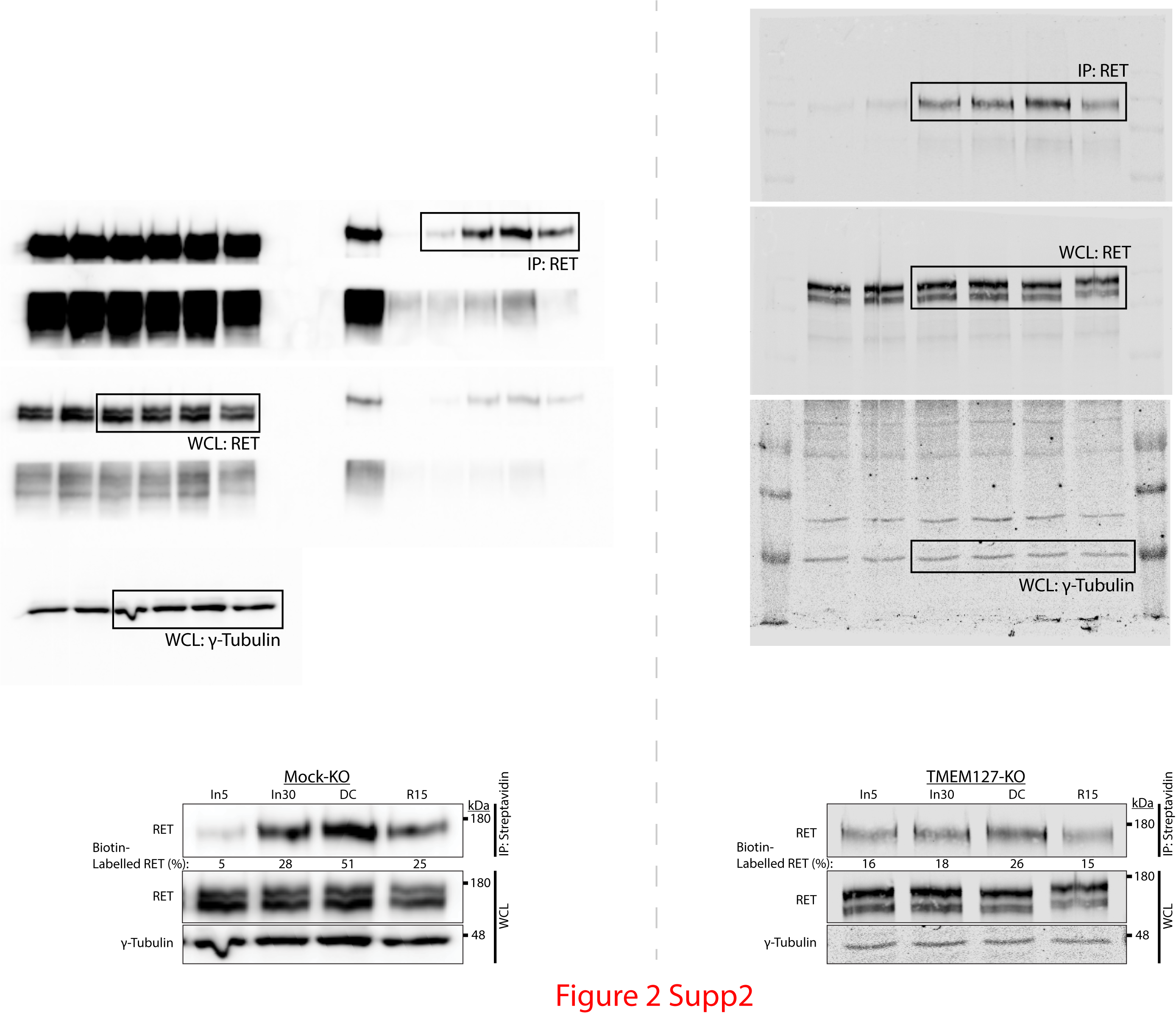

Supplement: Figure 2—figure supplement 2—source data 3. [file elife-89100-fig2-figsupp2-data3.zip › Figure 2 Supplement 2 - Source Blots.tif]

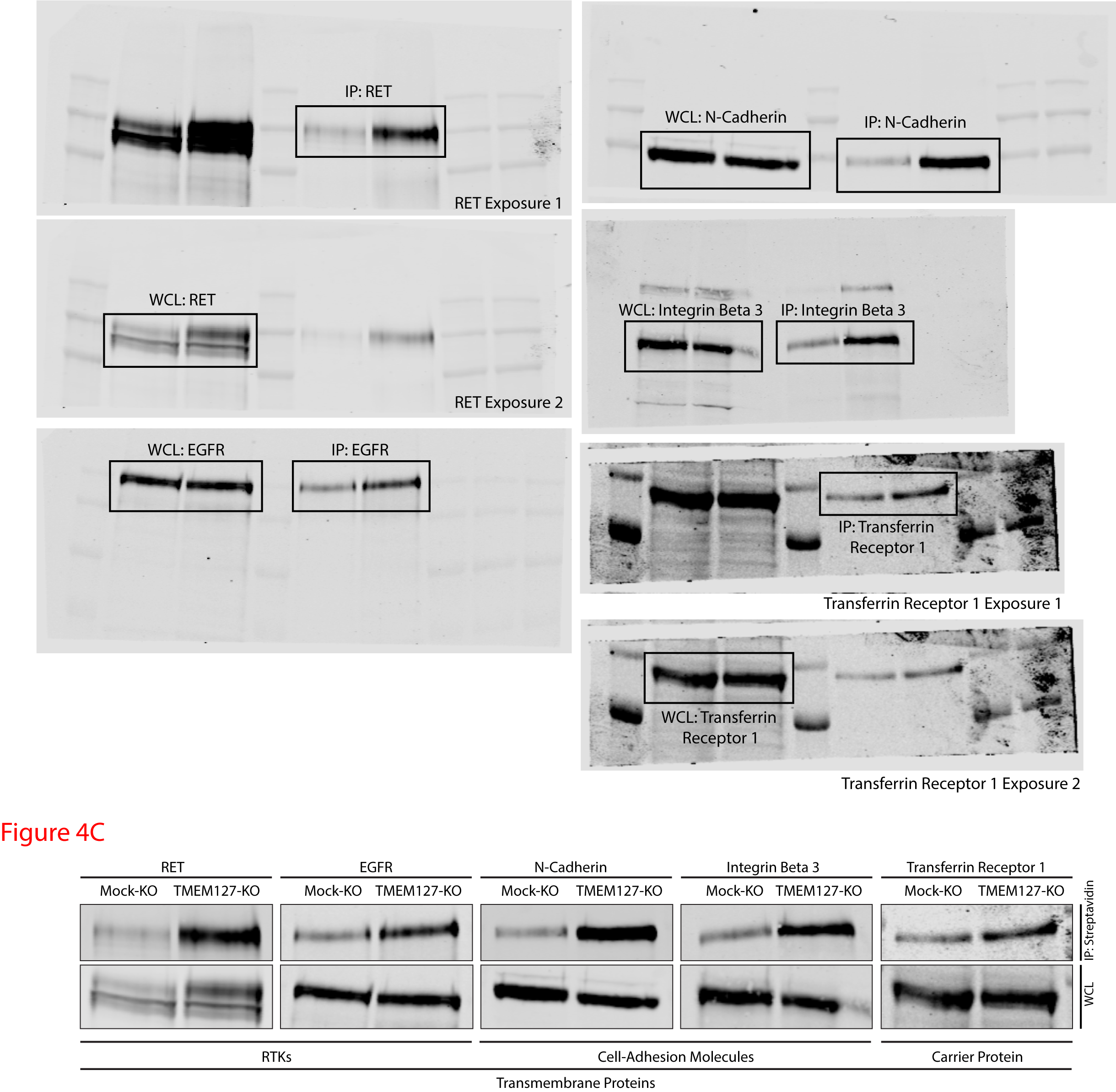

Supplement: Figure 4—source data 3. [file elife-89100-fig4-data3.zip › Figure 4 - Source Blots.tif]

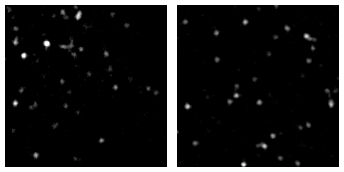

Supplement: Supplementary file 16 [file elife-89100-video1.gif]

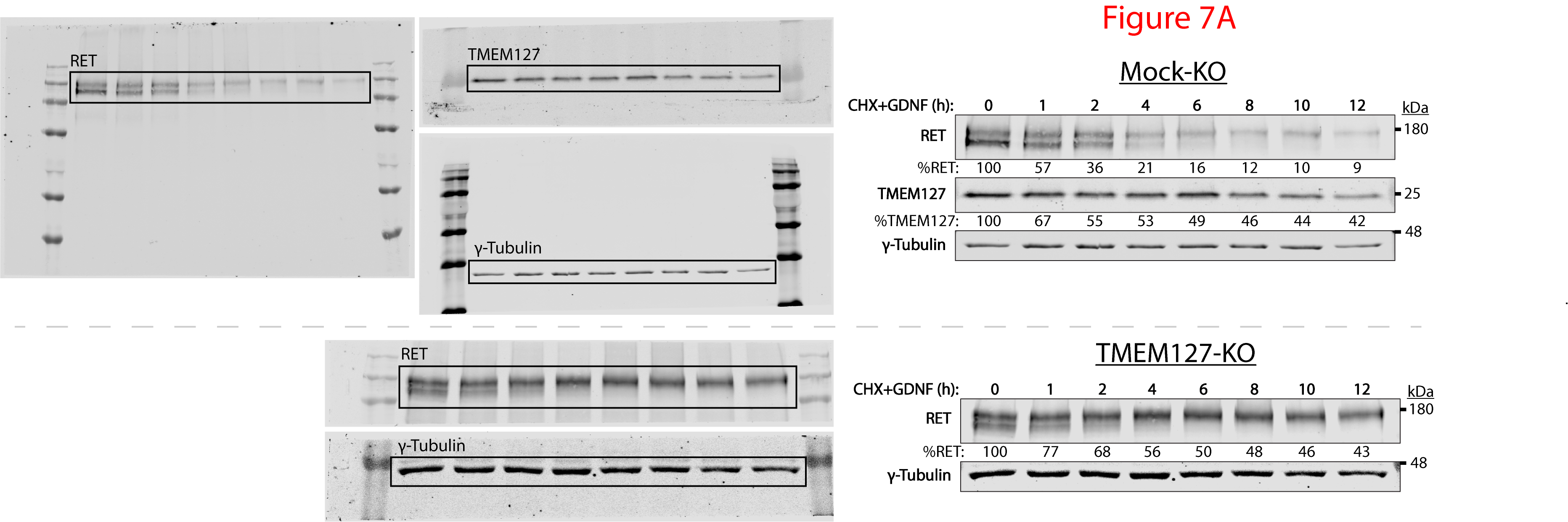

Supplement: Figure 7—source data 3. [file elife-89100-fig7-data3.zip › Figure 7 - Source Blots.tif]

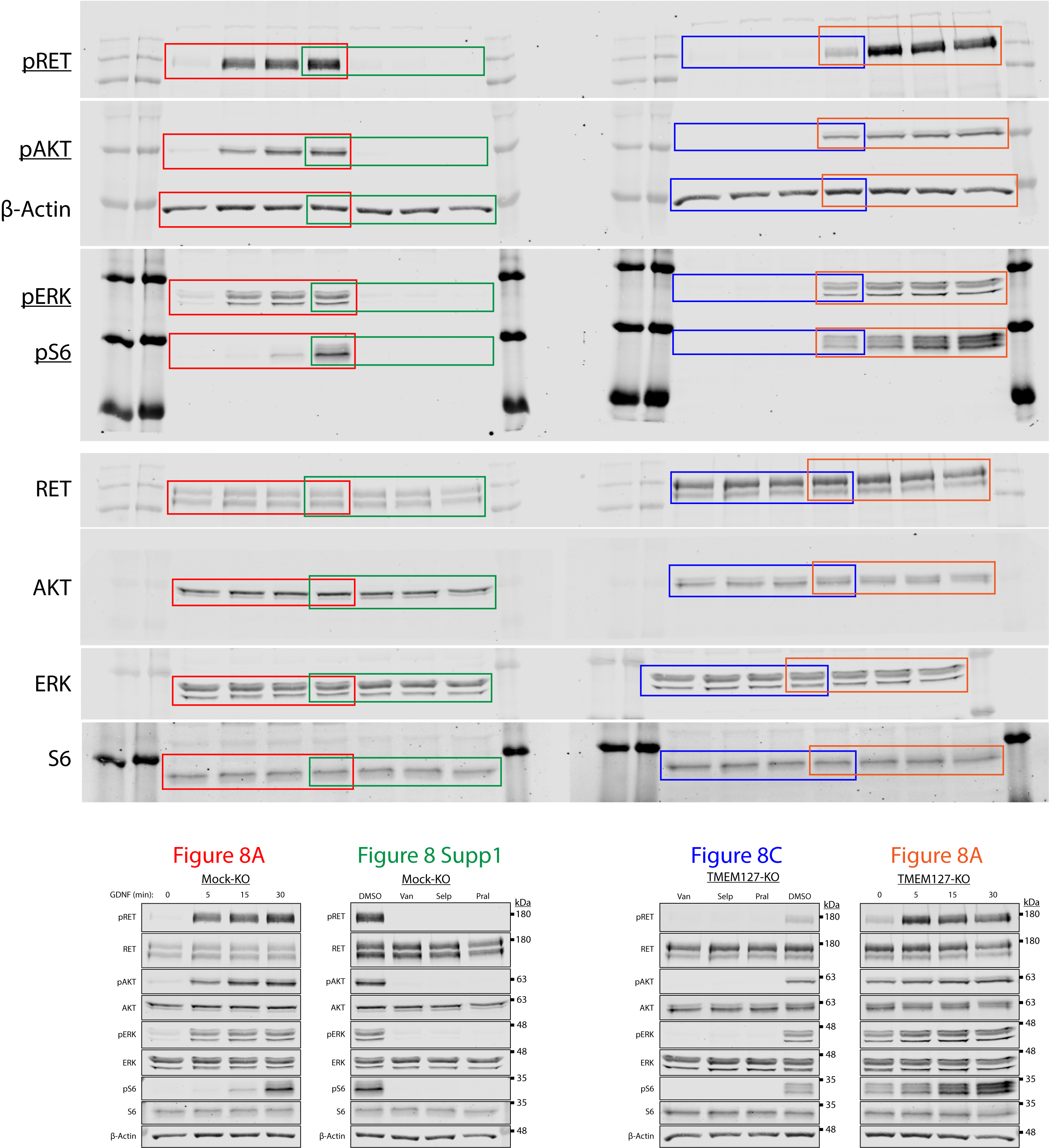

Supplement: Figure 8—figure supplement 1—source data 2. [file elife-89100-fig8-figsupp1-data2.zip › Figure 8 and Supplement 1 - Source Blots.tif]

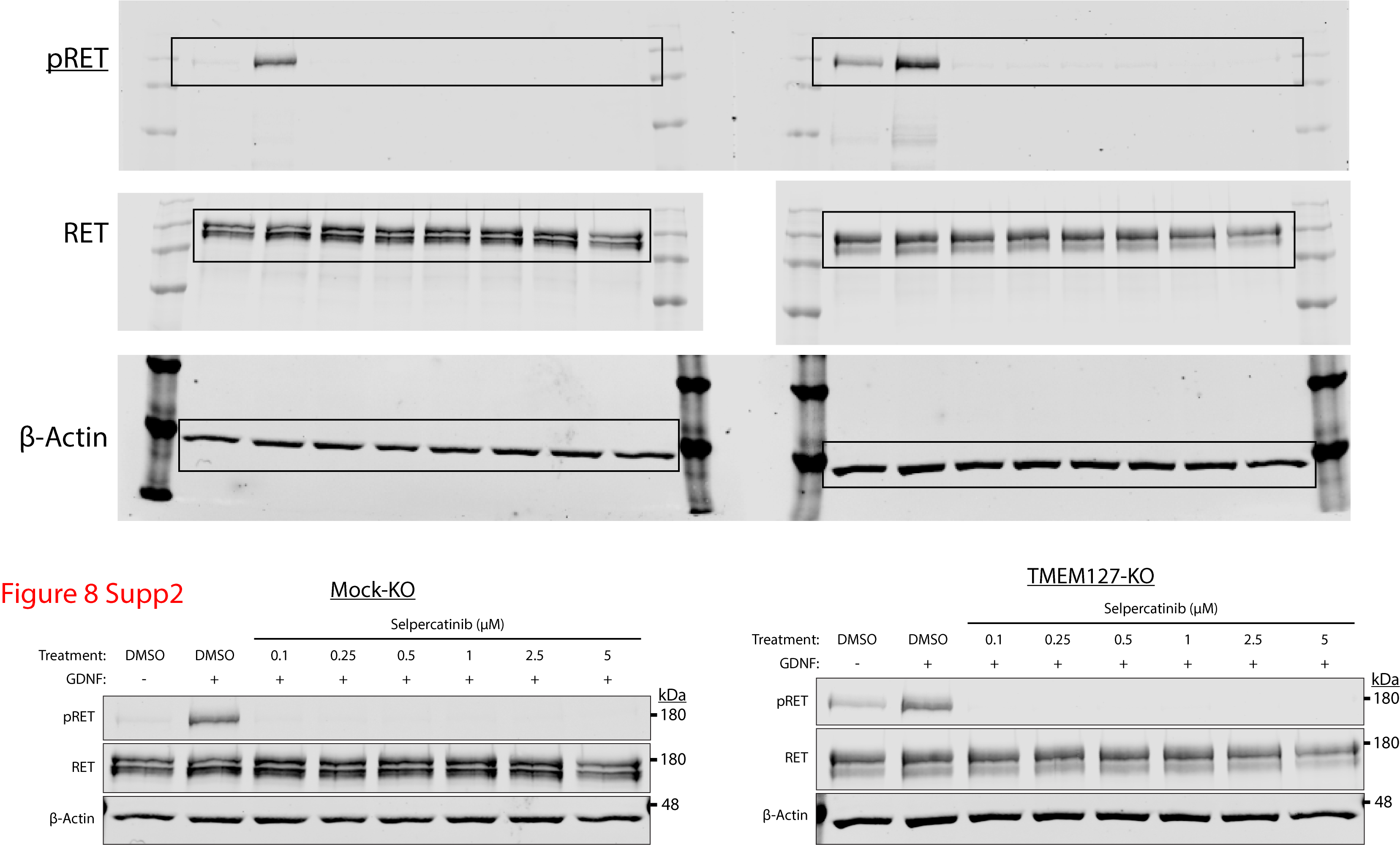

Supplement: Figure 8—figure supplement 2—source data 2. [file elife-89100-fig8-figsupp2-data2.zip › Figure 8 S2 - Source Blots.tif]
